# Functional significance of complex fluctuations in brain activity: from resting state to cognitive neuroscience

**DOI:** 10.3389/fnsys.2014.00112

**Published:** 2014-06-11

**Authors:** David Papo

**Affiliations:** Computational Systems Biology Group, Center for Biomedical Technology, Universidad Politécnica de MadridMadrid, Spain

**Keywords:** scaling, multifractals, ageing, weak ergodicity breaking, symmetry, fluctuation-dissipation theorem, cognitive neuroscience, resting state

## Abstract

Behavioral studies have shown that human cognition is characterized by properties such as temporal scale invariance, heavy-tailed non-Gaussian distributions, and long-range correlations at long time scales, suggesting models of how (non observable) components of cognition interact. On the other hand, results from functional neuroimaging studies show that complex scaling and intermittency may be generic spatio-temporal properties of the brain at rest. Somehow surprisingly, though, hardly ever have the neural correlates of cognition been studied at time scales comparable to those at which cognition shows scaling properties. Here, we analyze the meanings of scaling properties and the significance of their task-related modulations for cognitive neuroscience. It is proposed that cognitive processes can be framed in terms of complex generic properties of brain activity at rest and, ultimately, of functional equations, limiting distributions, symmetries, and possibly universality classes characterizing them.

## Introduction

Ideally, cognitive psychology aims at providing a description of the space of cognitive processes, the nature of each of them, and the way they interact. Cognitive processes are unobservable regimes of an underlying dynamical system. However, they can be reconstructed by considering that sequences of observable quantities, sampled during the execution of controlled cognitive tasks, are the output of this system.

In behavioral studies, the underlying system is construed as a black box function, with given tasks, supposed to summon given cognitive processes, as inputs, and observable behavioral performance as outputs.

Typically, a quantitative description of cognitive processes consists in calculating means and standard deviations of trial-averaged performance measures, implicitly assuming an underlying Gaussian distribution (which is completely described by its first two moments), and statistical independence of the various trials.

However, the results of numerous behavioral studies [see (Kello et al., 2010) for a review] cannot be reconciled with Gaussian distribution functions. Power-law distributions and temporal scaling have consistently been found for relatively short time series (~10^2^–10^3^ time points) (Gilden, 2001) of inter-trial fluctuations in performance levels, although finer temporal scales have also been considered, particularly for motor tasks (Cabrera and Milton, 2002; Diniz et al., 2011).

Behavioral scaling laws contain important information about cognitive function, viz. on how (non observable) components of cognition interact (Holden et al., 2009). For instance, power-law scaling of trial-to-trial performance variations has been taken to arise from multiplicative interactions among interdependent processes, suggesting that the mechanisms through which processes interact to give rise to cognitive performance may be no less fundamental than single components' functioning principles (Holden et al., 2009; Ihlen and Vereijken, 2010).

The scaling properties appear to be modulated in a task-specific way. For example, increasing task difficulty accelerates the transition from 1/f to white noise in decision-making time series (Correll, 2008; Grigolini et al., 2009).

Cognitive function is naturally understood as originating from brain activity, and quantitatively characterized in terms of the brain properties associated with the execution of given cognitive tasks. Cortical activity adds spatial and temporal scales unavailable in behavioral studies, so that scaling can be assessed within single process realizations.

The brain generates fluctuations with complex scaling properties (Novikov et al., 1997; Linkenkaer-Hansen et al., 2001; Gong et al., 2002, 2007; Freeman et al., 2003; Bianco et al., 2007; Suckling et al., 2009; Freyer et al., 2009), even in the absence of exogenous perturbations or changes in parameters controlling its activity. Only few experimental studies (Linkenkaer-Hansen et al., 2004; Popivanov et al., 2006; Buiatti et al., 2007; Bhattacharya, 2009; He et al., 2010; Ciuciu et al., 2011, 2012; Zilber et al., 2012) investigated the scaling properties of *task-related* brain activity, or their relationship with behavioral ones (Monto et al., 2008; Palva et al., 2013; Kello, 2013).

The aetiology and functional meaning of brain fluctuation scaling have been discussed at length. For example, the presence of spatial and temporal inverse-power law correlations is often taken to suggest that the brain lives near a second order phase transition, a condition optimizing information processing and storage, and dynamic response (Chialvo, 2010).

Here, instead, we discuss ways in which fluctuation properties can be used as metrics making cognitive function observable.

## A random walk around brain activity's space

To garner a physical intuition of the meaning of brain fluctuations one can think of brain activity as the motion of a random walker making steps of size *x* at given times *t*, or, in the continuous limit, of a diffusing macroscopic particle in a complex high-dimensional space, subject to viscous friction, with a time scale τ_*m*_, and driven by an additive random force with a characteristic time τ_η_ (Hsu and Hsu, 2009).

The relationship between τ_*m*_ and τ_η_ determines how the system evolves in this complex space, including traveled distances, velocity, degree to which the space is visited, time to reach a given target point, system's memory of its own trajectory within the landscape, relationship between spontaneous and task-related activity, and ultimately how microscopic fluctuations renormalize to give rise to observable macroscopic statistical properties (Papo, 2013b).

If spontaneous fluctuations were *Markovian*, with Gaussian δ-correlated noise, and τ_η_ « τ_*m*_, the particle would undergo normal diffusion: the step length would be taken from the Maxwell-Boltzmann equilibrium distribution, and the mean-square distance (MSD) traveled by the particle would scale linearly with time 〈|*x(t)*|^2^〉 ~ *t*. Under general conditions, the first passage time from a prescribed phase space domain would be characterized by a universal distribution, independent of the jump length distribution (Sparre Andersen, 1953). For *t* » τ_*m*_, the temporal autocorrelation of velocity fluctuations would behave as *C*(τ) ~ *exp*(−*t*/τ_*m*_), with a unique *characteristic time* τ_*m*_. The dynamics would hop without memory from one configuration to another, eventually visiting the whole phase space.

However, the properties of observed brain fluctuations are inconsistent with the Markovian approximation (Fraiman and Chialvo, 2012). Spontaneous fluctuations show temporal and spatial scale-free statistics (Novikov et al., 1997; Linkenkaer-Hansen et al., 2001; Gong et al., 2002; Stam and de Bruin, 2004; Expert et al., 2010; van de Ville et al., 2010). The MSD scales as 〈 |*x(t)*|^2^〉 ~ *t*^2ν^ with ν ≠ 1/2, so that its diffusion is *anomalous*, and indeed even *strongly* anomalous (Suckling et al., 2009; Ciuciu et al., 2011, 2012; Zilber et al., 2012), with the *q-th* moments scaling as 〈|*x(t)*|^*q*^〉 ~ *t*^*q*ν(*q*)^, with ν(*q*) ≠ const (Castiglione et al., 1999). Appropriately rescaled average temporal fluctuations collapse onto *universal scaling functions* (Sherrington, 2010; Friedman et al., 2012; Shriki et al., 2012).

Exponential relaxation is replaced by complex scaling, e.g., of a Mittag-Leffler type (Bianco et al., 2007), with stretched exponential relaxation at microscopic scales (*t* < τ), and inverse power-law scaling *C*(τ) ~ τ^−α^, for *t* » τ, so that, for α ≤ 1, the *correlation time* τ_*C*_ = ∫^ξ^_0_*C(t)dt* diverges, leading to a scale-free process with memory. The system undergoes *ageing* (Bianco et al., 2007): correlations are time-inhomogeneous, with a dependence on the time of application of a given field, history-dependent (Sherrington, 2010), and weakly non-ergodic (Bianco et al., 2007), as some phase space region may take extremely long times to be visited (Bouchaud, 1992).

Activity shows statistical and dynamical *intermittency*: on the one hand, although large-scale fluctuations are approximately Gaussian, non-Gaussian fluctuations appear at higher frequencies (Freyer et al., 2009). On the other hand, activity is characterized by alternating laminar and turbulent phases (Gong et al., 2007; Allegrini et al., 2010, 2011).

## Understanding brain fluctuations

The statistical and dynamical properties of brain fluctuations contain information on the structure of the functional space within which brain dynamics evolves, and on the style, as it were, with which brain dynamics explores its dynamic repertoire (Ghosh et al., 2007; Deco et al., 2011; Betzel et al., 2012).

### From single steps to complete walks

Scaling laws indicate that the walker takes steps of all sizes, from local to extremely long jumps.

More importantly, probability distributions contain information on how observable large-scale outcomes arise from the interactions of many small-scale processes (Frank, 2009). Observed probability distributions can be thought of as reproducible macroscopic features emerging from the sum of highly fluctuating individual elements. It is natural to see this sum as representing the temporal aggregation of fluctuations within a given time-window.

The central limit theorem (CLT) ensures that the limit distribution of the sum of a large number of random variables is a stable law. The law is Gaussian if the variables are independent and have finite variance. For correlated or infinite variance fluctuations, the CLT ought to be generalized, and the stable law is not Gaussian but Lévy. Importantly, in the latter the largest term is of approximately the same order of magnitude of the sum, indicating that extreme events dominate the underlying process (Laguës and Lesne, 2008).

From a dynamical view-point, the CLT accounts for normal diffusion and the time dependence of the MSD (or the walker's position), while generalized CLTs result in anomalous diffusion, which differs both in relaxation speed, and in the probability distribution's shape, even at very long times.

Probability distributions can be seen as resulting from the iteration of some action on them. For instance, stable laws are fixed points of the convolution operation. Somehow equivalently, fluctuation distributions can be understood as asymptotic behaviors emerging as the system is coarse-grained and rescaled (Hochberg and Pérez-Mercader, 2003). Scale-free distributions are fixed points of a renormalization flow, and universality classes are their basins of attraction. The surface comprising the models flowing into the same fixed point separates the space into phases, corresponding to different macroscopic phenomenologies (Laguës and Lesne, 2008). Universality can be understood in terms of relevant and irrelevant operators, depending on the consequence they have on the statistical behavior (Laguës and Lesne, 2008).

Probability distributions can also be seen as solutions to specific problems expressed e.g., by differential equations (Barenblatt and Zel'dovich, 1972). For instance, probability distributions are solutions of the Fokker-Planck equation of evolution of the particle's transition probabilities, under given information constraints (Jaynes, 1957). For the linear diffusion equation, the solution is a time-evolving spatial Gaussian probability function maximizing the Shannon entropy. Correlated anomalous diffusion is governed by a nonlinear Fokker-Planck equation whose exact stationary solutions are probability distributions maximizing Tsallis generalized entropy (Borland, 1998).

### Emergence of structure: memory, temporal order, and non-locality

Correlations are propagators, whose characteristic length ξ constitutes an active time window within which all points are somehow related to each other.

A Markovian system has perfectly elastic almost instantaneous relaxation and no memory: the time axis tends to be infinitely fragmented, so that activities of overall duration *L* are *temporally disordered* (*L* » ξ).

Brain fluctuations' loss of scale separation allows microscopic randomness to renormalize and become macroscopically detectable (Grigolini et al., 1999): correlated driving noise and cross-scale relationships produce *temporally ordered* structures (*L* ~ ξ), so that activity at a given time point is temporally *non-local*, and not easily divorced from that occurring within the scaling range.

With temporal scaling, fluctuations no longer have a characteristic time; more than to a multiplicity of scales {τ_*i*_}, the emphasis shifts to some *relationship* between them. The brain's functional heterogeneity introduces a spatial distribution of time scales {σ_*i*_} inducing a structure 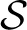. Eventually, the studied dynamics is a field ϕ($\vec{s}$, *t*) ∈ **Φ**, where **Φ** = {ϕ} is a space of systems, endowed with a spatio-temporal structure {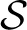 * ℜ}, with arbitrarily complex topological properties (Zaslavsky, 2002), and which can become observable through a wealth of collective state variables *X* ∈ **X**.

The structure {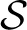 * ℜ} is a dynamical system in the space of fields ϕ, relating representations of the process at different scales (Friedrich et al., 2000; Bacry et al., 2001; Longo et al., 2012). For instance, at any given scale λ within the scaling range, the probability *P*(*x, t*) that the particle traveled a distance *x* at time *t* can be thought of as the convolution of the distribution *P*_Λ_(*x, t*) at the coarsest scale Λ and a probability distribution *G*(.), not necessarily a power-law (Chainais et al., 2005), expressing the relationships across time scales (Castaing et al., 1990). For scale-invariant processes, *P*(*x, t*) = *t*^−ν^
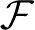
(*x/t*^ν^), *G* collapses into a single point, and is simply the *scaling exponent* ν. Scale invariance breakdown indicates that *P*(*x, t*) is specified by a complex spectrum of scaling exponents.

The set of renormalization operators is endowed with some structure, e.g., a multiplicative semi-group structure, and a covariance property comparable to that of tensors under the action of rotations, with scale invariance replacing Galilean invariance and fractal geometry the Euclidean one (Lesne, 2008a). In turn, scaling laws can be seen as the statistical properties prescribed by the symmetries of a (semi)group on the time-scale space (Borgnat et al., 2003).

Altogether, the presence of complex fluctuations allows treating brain activity as a physical object, defining subparts, and relationships among them, and ultimately using theoretical physics tools such as functional analysis and algebra to characterize them.

### Velocity and operational time

The presence of scaling can be interpreted in dynamical terms in various ways.

Furthermore, the Lamperti transform establishes a bijective correspondence between self-similar processes on ℝ^+^ and stationary processes on ℝ (Flandrin et al., 2003). Self-similar solutions reflect a uniform propagation regime (Barenblatt and Zel'dovich, 1972), and the system can be seen as moving at constant velocity, given by the scaling exponent (Sornette, 2004), whereas the breakdown of exact self-similarity indicates that the propagator is not time-stationary.

The scaling properties also define an intrinsic time of the process. This can be seen by considering that the random walk of brain activity has a waiting-time distribution (WTD) between jumps scaling as a power-law. The WTD defines an internal *operational time*, which can grow sub- or super-linearly with *physical time* (Sokolov and Klafter, 2005). Without multiplicative interactions, *operational* and *physical* time coincide. Multiplicative cross-scale interactions bias the WTD so that, local probability densities become time-dependent and *intermittent*, and time translational invariance is broken (Crisanti and Ritort, 2003). The observed Mittag-Leffler fluctuation distribution (Bianco et al., 2007) may in fact stem from the process intermittent subordination with internal time.

## Dynamical regimes and fluctuation dissipation relations

Brain fluctuation properties help relating two only seemingly antagonistic aspects of brain activity: spontaneous and task-induced brain activity. For Gaussian δ-correlated fluctuations, the fluctuation-dissipation theorem (FDT) ensures that the system's integrated response χ(*t, t*′) at time *t* to an external field applied at time *t*′ and the autocorrelation function *C*_*X*_(*t*, *t*′) of the unperturbed system are linked by the temperature *T* of the bath with which the system is in equilibrium (Kubo, 1966). Translated in terms of brain activity, the FDT would establish an equivalence between stimulus-evoked and spontaneous brain fluctuation correlations (Papo, 2013c).

Complex multiscale fluctuations suggest that thermalization happens simultaneously at widely different timescales, so that the FDT in its classical form is not expected to hold (West et al., 2008). For systems with the type of intermittency observed for brain activity, the linear response is anomalous even with simple stimuli (Silvestri et al., 2009; Allegrini et al., 2010). The way the FDT is violated and the ingredients necessary to recover it can be used in various ways as descriptors of brain activity.

First, the properties of ongoing fluctuations define the form of the generalized FDT holding for brain activity and, *in fine*, the way stimulus information is transferred to the brain. The presence of correlated noise affects the particle's transport properties and corresponding dynamics (Machura and łuczka, 2010), and information transfer is maximized when stimuli and brain fluctuations display similar scaling properties (Allegrini et al., 2007; West and Grigolini, 2010; Aquino et al., 2011). Moreover, scaling exponents mark dynamical transitions between qualitatively different response regimes (Burov and Barkai, 2008).

Second, the nature of FDT violation helps understanding at what scales correlations and memory start playing a role, and correctly characterizing the underlying dynamics by specifying the additional degrees of freedom necessary to recover Markovianity (Zwanzig, 2001).

Finally, *effective temperatures*, i.e., what a thermometer responding on the time scale at which the system slowly reverts to equilibrium would measure, which may be used to derive a generalized FDT (Cugliandolo et al., 1997), constitute intrinsic time scales of the system. Fluctuations ultimately identify a spatial distribution of scale-dependent relationships between spontaneous and stimulus-induced brain activity, quantifying the extent to which each scale deviates from equilibrium (Papo, 2013a). This reflects the fact that a path realizes qualitatively different diffusion processes at different temporal and the spatial scales.

## Task-related modulations

Because most complex scaling properties are presumably generic, psychologists are primarily interested in the extent to which cognitive activity may affect them. Furthermore, precisely because they are generic, task-induced modulations of these properties represent powerful descriptors of the underlying processes.

## Cross-overs and symmetry changes

Numerous cognitive tasks have been shown to modulate the scaling exponents of brain fluctuation probability functions (Linkenkaer-Hansen et al., 2004; Popivanov et al., 2006; Buiatti et al., 2007; He et al., 2010; Ciuciu et al., 2011, 2012; Zilber et al., 2012). Task demands also appear to enhance data collapse and universality of brain fluctuations (Bhattacharya, 2009).

Cognitive demands may push brain activity toward the basin of attraction of adaptively advantageous probability distributions. Cognitive function would be tantamount to designing a driving noise function making the system's stationary distribution equal a desired target one. Moreover, insofar as power laws are solutions of functional equations, rather than frequency or amplitude modulators, cognitive processes may be conceptualised as operators acting upon the functional form of brain activity.

A still poorly explored possibility is that these modulations represent cross-overs between universality classes. This would allow classifying observed cognitive function as operators acting on symmetries (Lübeck, 2004). Renormalization flows would represent generalized dynamic pathways within the functional space, and universality classes a partition of this space, quantifying robustness with respect to control parameter variations (Lesne, 2008a,b).

Whether and how cognitive demands act on brain activity's symmetries is a deserving matter (Freeman and Vitiello, 2006; Buice and Cowan, 2009). For instance, the transition from mono- to multifractal distributions has been reported at the late stages of various fracture phenomena (de Arcangelis and Herrmann, 1989; Kapiris et al., 2004). However, whether spontaneous activity is temporally scale-free (van de Ville et al., 2010) or breaks down scale invariance (Ciuciu et al., 2011, 2012; Wink et al., 2012; Zilber et al., 2012) is still an open debate. Existing discrepancies may stem from the order parameter used to evaluate scaling, e.g., whether it is local or has prominent spatial extension, as heterogeneity and disorder may directly affect the scaling exponents.

The shrinking of the multifractal spectrum associated with performance of cognitive tasks may amount to selecting a set of complex patterns from the available repertoire, or to modifying the rate at which these patterns are re-edited across the system (Kenet et al., 2003; Betzel et al., 2012). On the other hand, stimuli drive neural activity away from criticality (Kohen-Kashi Malina et al., 2013), an action reminding the interruption of ageing caused by an external field forcing a glassy material (Kranz et al., 2010). In this sense, one may interpret multifractality as a sign of ageing (Allegrini et al., 2004).

## Steering within the phase space

As they modulate the temporal scaling of fluctuations, cognitive demands affect the temporal organization of brain activity and the corresponding operational time.

The observable outcome could come in the form of a modulation of cross-over scales, e.g., the time scales at which fluctuations start converging to a Gaussian distribution, varying the likelihood of large scale events (Mantegna and Stanley, 1994), the length interval over which activity can be considered a Markov process, the time scale of the transition from microscopic to macroscopic dynamics (Aquino et al., 2007), or the degree of non-ergodicity, corresponding to different ways of visiting the state space (Lomholt et al., 2013).

Furthermore, stimulus-induced modulations of temporal correlations may induce phase transitions in first-passage times (Carretero-Campos et al., 2012) and in response regimes (Burov and Barkai, 2008), and may influence fluctuations' transition to scaling, while endogenous activity likely affects the WTD scaling properties (Aquino et al., 2011).

Finally, cognitive demands may bias either the probabilities or the occurrence times of the walker's jumps (Allegrini et al., 2004), and therefore the operational time associated with a given process.

## Conclusions

We addressed the question of whether and how brain fluctuations help describing non observable cognitive processes.

That observed behavior is a product of brain activity is a matter of general consensus. Here, we further proposed that the former can be described in terms of the generic properties of the latter, such as scaling regimes and their basins of attraction, symmetries (not only scale invariance), FDT violations. Ultimately, it is tempting to conceive of observed behavior as a macroscopic property emerging from the renormalization of microscopic brain fluctuations.

Such characterization of the action of cognitive demands on brain activity affords a wealth of order parameters through which activity becomes observable, each representing a cut, in different dimensions and scales, of the same underlying space. More generally, it allows a conceptualization whereby cognitive processes operate upon the structure of brain activity, producing effects observable from various perspectives (e.g., structural or dynamical). Eventually, this shapes a functional space for which internal structure, and transition and combinatory rules can be extracted.

Finally, it is important to warn that these descriptions do not unambiguously characterize the aetiology of fluctuation properties, as similar scaling properties may stem from qualitatively different generators (Magdziarz et al., 2009; Meroz et al., 2013; Thiel et al., 2013) which may be difficult to distinguish with a finite amount of data (Grigolini, 2008).

### Conflict of interest statement

The author declares that the research was conducted in the absence of any commercial or financial relationships that could be construed as a potential conflict of interest.
